# Intact interleukin-10 receptor signaling protects from hippocampal damage elicited by experimental neurotropic virus infection of SJL mice

**DOI:** 10.1038/s41598-018-24378-z

**Published:** 2018-04-17

**Authors:** Ann-Kathrin Uhde, Malgorzata Ciurkiewicz, Vanessa Herder, Muhammad Akram Khan, Niko Hensel, Peter Claus, Michael Beckstette, René Teich, Stefan Floess, Wolfgang Baumgärtner, Klaus Jung, Jochen Huehn, Andreas Beineke

**Affiliations:** 1 Department of Pathology, University of Veterinary Medicine Hannover, Hannover, Germany; 2 Center for Systems Neuroscience, Hannover, Germany; 3 Department of Pathobiology, Faculty of Veterinary & Animal Sciences, PMAS–Arid Agriculture University, Rawalpindi, Pakistan; 4 Institute of Neuroanatomy and Cell Biology, Hannover Medical School, Hannover, Germany; 5 Niedersachsen-Research Network on Neuroinfectiology (N-RENNT), Hannover, Germany; 6 Experimental Immunology, Helmholtz Centre for Infection Research, Braunschweig, Germany; 7 Institute for Animal Breeding and Genetics, University of Veterinary Medicine Hannover, Hannover, Germany

## Abstract

Theiler’s murine encephalomyelitis virus (TMEV) infection represents an experimental mouse model to study hippocampal damage induced by neurotropic viruses. IL-10 is a pleiotropic cytokine with profound anti-inflammatory properties, which critically controls immune homeostasis. In order to analyze IL-10R signaling following virus-induced polioencephalitis, SJL mice were intracerebrally infected with TMEV. RNA-based next generation sequencing revealed an up-regulation of *Il10*, *Il10rα* and further genes involved in IL-10 downstream signaling, including *Jak1*, *Socs3* and *Stat3* in the brain upon infection. Subsequent antibody-mediated blockade of IL-10R signaling led to enhanced hippocampal damage with neuronal loss and increased recruitment of CD3^+^ T cells, CD45R^+^ B cells and an up-regulation of *Il1α* mRNA. Increased expression of *Tgfβ* and *Foxp3* as well as accumulation of Foxp3^+^ regulatory T cells and arginase-1^+^ macrophages/microglia was detected in the hippocampus, representing a potential compensatory mechanism following disturbed IL-10R signaling. Additionally, an increased peripheral *Chi3l3* expression was found in spleens of infected mice, which may embody reactive regulatory mechanisms for prevention of excessive immunopathology. The present study highlights the importance of IL-10R signaling for immune regulation and its neuroprotective properties in the context of an acute neurotropic virus infection.

## Introduction

*Theiler’s murine encephalomyelitis virus* (TMEV), a neurotropic picornavirus, preferentially targets limbic and temporal structures, including the hippocampus, during acute infection in mice^1,2^. Due to robust antiviral immunity, C57BL/6 mice are able to eliminate the virus but develop marked hippocampal damage with neuronal loss, which is associated with seizure development^3–5^. In addition to acute neurological symptoms, it has been shown that TMEV-infection also leads to increased chronic seizure susceptibility, making *Theiler’s murine encephalomyelitis* (TME) a valuable infectious epilepsy model^2^. Hippocampal neuronal damage is further associated with impaired cognitive ability, anxiety-like behavior and disrupted spatial memory of infected C57BL/6 mice^6,7^. By contrast, SJL mice develop a biphasic disease with TMEV persistence and spinal cord demyelination due to ineffective antiviral immunity^8^. Unlike C57BL/6 mice, TMEV-infected SJL mice show a subclinical, transient polioencephalitis along with mild neuronal degeneration, which is not accompanied by seizure development in the acute disease^5^.

IL-10 is a pleiotropic cytokine with profound anti-inflammatory and tolerogenic properties, which is produced by resident microglia, CNS-infiltrating macrophages and lymphocytes, particularly regulatory T cells (Treg), in inflammatory disorders^9^. It is crucial for maintenance of immune homeostasis and plays a central role in a variety of human diseases^10–12^. Immunomodulatory effects following IL-10R ligation are mediated by activation of the *Stat3* pathway and *Socs3*^13^. In contrast to the predominantly therapeutic effect of IL-10 in autoimmune diseases, such as experimental autoimmune encephalomyelitis (EAE)^10^, an ambivalent and more complex function of IL-10R signaling has been described in infectious CNS disorders. Here, both beneficial and detrimental effects can be observed. On the one hand, IL-10 protects from excessive immune responses by downregulating self-destructive inflammatory processes^14,15^. On the other hand, overexpression of IL-10 is known to cause uncontrolled pathogen replication and increased pathogen mediated lesions^14,16^. For instance, IL-10 contributes to T cell exhaustion and causes persistence of *lymphocytic choriomeningitis virus* (LCMV) in C57BL/6 mice, which is circumvented by treatment with IL-10R blocking antibodies^17,18^. Similarly, genetic and antibody-mediated blockade of IL-10 signaling enhances antiviral immunity and decreases mortality rates in murine *West Nile Virus* infection^19^. By contrast, IL-10-deficiency in mice infected with neurotropic mouse hepatitis virus augments immune mediated brain damage without having any impact on the virus load^15^. Moreover, several studies have challenged the perception of IL-10 solely as an immunosuppressive molecule, since opposing effects on different Treg subsets and stimulating effects on effector T cells have been described depending on their activation state^20–24^.

Our previous studies on IL-10 in TME revealed only limited effects of anti-IL-10R treatment on spinal cord lesions and virus load in SJL mice during the chronic disease^25^. In acute TME, an elevated *Il10* expression primarily by infiltrating T cells was observed in the brain of SJL mice compared to those of seizure-prone C57BL/6 mice^26^. However, whether enhanced IL-10 signaling exhibits neuroprotective properties by preventing an excessive inflammatory response and/or accounts for reduced antiviral immunity during early infection has not yet been elucidated in TMEV-infected SJL mice^27^. Therefore, the aim of the present study was (i) to perform an expressional analysis of IL-10R signaling during the course of TMEV-induced polioencephalitis in SJL mice and (ii) to determine the effects of IL-10R blockade on hippocampal pathology during early TME in SJL mice.

## Materials and Methods

### Experimental design

25 five-week old female SJL and 5 five-week old female C57BL/6 mice (Harlan Winkelmann) were inoculated with 1.63 × 10^6^ PFU of TMEV (BeAn-strain, 0 days post infection [dpi]) into the right cerebral hemisphere following general anesthesia, as described previously^25^. In the first experiment, necropsy was performed in groups of five SJL animals at 4, 7 and 14 dpi, respectively. After euthanasia, animals were perfused via the left ventricle of the heart with PBS and brains were removed immediately. Subsequently, cerebra were cut transversally at the level of optic chiasm and the caudal parts were fixed in 10% formalin for 24 h, embedded in paraffin wax and processed for histology and immunohistochemistry (IHC). The rostral parts were snap frozen and stored at −80 °C until use for RNA extraction and RNA-Seq. In a second animal experiment, the effect of IL-10R signaling on immune regulation and neuropathology in TME was determined by Ab-mediated receptor blockade in SJL mice. Results were compared to TMEV-infected C57BL/6 mice not receiving IL-10Rα-specific Ab. SJL mice were infected as described above and injected with 250 µg rat anti-mouse IL-10Rα-specific Ab (clone: 1B1.3 A, BioXCell) or rat IgG1-specific isotype control (BioXCell) intraperitoneally at 0 dpi, respectively. The antibody clone has been shown to effectively block IL-10R in mice and induce immunopathology mediated by disturbed IL-10 signaling^17,19,25,28^. At 7 dpi, animals were euthanized and perfused. Brain was sampled for histology, IHC, RT-qPCR and plaque assay. In addition, spleen tissue was taken for flow cytometry, IHC and targeted RT-qPCR screening and blood samples were collected for flow cytometry by addition of heparin (1:1000, Heparin-Natrium-5000-ratiopharm VR GmbH).

All experiments were conducted in accordance with German law for animal protection and with the European Communities Council Directive for the protection of animals used for experimental purposes. Approval and authorization of the animal experiment were given by the local authorities (Niedersächsisches Landesamt für Verbraucherschutz und Lebensmittelsicherheit (LAVES), Oldenburg, Germany, permission numbers: 509c-42502-02/589, 33.12-42502-04-13/1138 and 33.9-42502-04-11/0538).

### Immunohistochemistry

IHC was performed on serial cross sections of the hippocampus using a CD3-specific Ab for detection of T cells, a CD45R/B220-specific Ab for detection of B cells, an TMEV-specific Ab for visualization of TMEV-antigen^29^ and a Foxp3-specific Ab for recognition of Treg. Additionally, a CD107b-specific Ab and an arginase-1 (Arg1)-specific Ab for detection of macrophages/microglia were used^30^. Damaged axons were labeled with a β-amyloid precursor protein (β-APP)-specific Ab^31^ and mature neurons were marked with an Ab directed against the neuronal nuclear protein NeuN. For detection of macrophages in spleen tissue a Chitinase-3-like protein 3 (CHI3L3, syn. Ym1)-specific Ab was used. Expression of Arg1 and CHI3L3 are induced by Th2 cytokines and considered to be associated with anti-inflammatory properties of macrophages/microglia^32,33^. All reactions were conducted as previously described^25,34,35^ and summarized in Table 1.

**Table 1:** Summary of Ab used for immunohistochemistry.

Ab	Clonality/clone	Manufacturer/Order No.	Pre-treatment and dilution	Specifity
CD3	pc	DakoCytomation A0452	Citrate buffer/microwave 1:1000	T cells
CD45R/B220	mc RA3-6B2	BD Biosciences 553085	Citrate buffer/microwave 1:1000	B cells
TMEV	pc	#	No pretreatment 1:2000	TMEV BeAn
Foxp3	mc FJK-16s	eBioscience 14-5773	Citrate buffer/microwave 1:50	Treg
CD107b	mc M3/84	AbD Serotec MCA2293B	Citrate buffer/microwave 1:200	Activated macrophages/microglia
Arginase-1	pc	Santa Cruz Biotechnology sc-18351	Citrate buffer/microwave 1:50	Macrophages/microglia with anti-inflammatory properties
β-APP	mc 22C11	Chemicon International MAB348	Citrate buffer/microwave 1:2000	Axonal damage
NeuN	mc A60	Chemicon International MAB377	Citrate buffer/microwave 1:2000	Postmitotic neurons
CHI3L3	pc	Abcam ab93034	Citrate buffer/microwave 1:100	Macrophages/microglia with anti-inflammatory properties

β-APP = Beta-amyloid precursor protein; CHI3L3 = chitinase-3-like protein 3; Iba-1 = ionized calcium binding adaptor molecule 1; mc = monoclonal; NeuN = neuronal nuclear protein; pc = polyclonal; TMEV = Theiler’s murine encephalomyelitis virus; # = not commercially available, published previously^29^.

For evaluating CD3, CD45R/B220, Foxp3, TMEV, CD107b, Arg1 and β-APP the absolute numbers of immunoreactive cells and axons, respectively, were manually counted in coronal sections of the hippocampus of each animal. The amount of NeuN^+^ neurons in the hippocampus was quantified on digitalized slides by densitometry using the analySIS® 3.2 software. CHI3L3^+^ cells in spleen sections were manually counted, averaging the sum of 10 randomly chosen high power fields per animal.

### Molecular analyzes

#### RNA isolation and reverse transcription

RNA was isolated from snap frozen brain and spleen tissue using an Omni´s PCR Tissue Homogenizing Kit (Süd-Laborbedarf GmbH), QIAzol^TM^ Lysis Reagent (Qiagen GmbH) and RNeasy^®^ Mini Kit (Qiagen GmbH) according to the manufacturer’s protocols. Subsequently, equal amounts of RNA were transcribed into cDNA with the Omniscript^TM^ RT Kit (Qiagen GmbH), RNAseOut^TM^ Recombinant Ribonuclease Inhibitor (Invitrogen^TM^ GmbH) and Random Primers (Promega GmbH) as described previously^35,36^.

#### RNA-based next generation sequencing (RNA-Seq)

Quality and integrity of RNA isolated from brain tissue was controlled on Agilent Technologies 2100 Bioanalyzer (Agilent Technologies). Purification of poly*-*A containing mRNA was performed using poly*-*T oligo attached magnetic beads (Illumina). Subsequently, mRNA was used for library preparation using the Script Seq v2 Library preparation kit (Illumina). Sequencing was carried out on Illumina HiSeq. 2500 using 50 bp single read. The sequenced libraries were assessed for read quality with *FastQC* (http://www.bioinformatics.babraham.ac.uk/projects/fastqc). Quality assessment showed neither insufficient read quality, nor nucleotide frequency biases introduced by primer contamination. Therefore, libraries were directly aligned to mouse reference genome (assembly: GRCm38) using splice junction mapper *Tophat2* v1.2.0^37^ with default parameterization.

Reads aligned to annotated genes were quantified with htseq-count (http://www-huber.embl.de/users/anders/HTSeq) program and RPKM (reads per kilobase max. transcript length per million mapped reads) normalized values were computed from raw gene counts. The principal component analysis (PCA) of the log2 transformed, scaled and mean centred RPKM values was performed using base functions *scale* and *prcomp* from the statistical data analysis framework R.

To determine the kinetics of *Il10* and related genes involved in IL-10 downstream signaling, six genes involved in interleukin-10 signaling were selected according to the Reactome pathway database^38,39^ (Pathway-Identifier: R-MMU-6783783) and literature research^40–42^. RPKM values of these genes were used for statistical analysis.

#### Real-time quantitative polymerase chain reaction (RT-qPCR)

RT-qPCR of brain tissue was performed for TMEV, *Il1α*, *Il2*, *Il4*, *Il5*, *Il6*, *Il10*, *Foxp3*, *Tnf*, *Ifnγ*, *Tgfβ1*, and three reference genes (*Gapdh*, *actβ*, *Hprt*) by use of the Mx3005P Multiplex Quantitative PCR System and Brilliant III Ultra-Fast SYBR^®^ Green RT-qPCR Master Mix (Agilent Technologies). All primer sequences were taken from the literature^25,26,35,43–45^ and are listed in supplemental Table S1. For quantification, ten-fold serial dilution standards ranging from 10^8^ to 10^2^ copies/µl were prepared. The normalization factor for correction of experimental variations was calculated from the three reference genes using geNorm software version 3.4^46^.

#### Targeted RT-qPCR screening

2.5 µg total RNA of each spleen was subjected to a reverse transcription reaction in a total volume of 40 µl employing 5 × FS-Buffer, 3 µg random hexamer primer, 200 U M-MLV transcriptase, 0.02 µmol dNTPs, and 0.4 µmol DTT (all Invitrogen^TM^ GmbH) as well as 40 U RNAse inhibitor (Agilent Technologies). 70 °C denaturation step was followed by a 1.5 h 42 °C reverse transcription step. 1 µl of individual cDNA preparations from each IL-10R-blocked and isotype-treated mouse, respectively, was pooled. The two pools were diluted 1:200 for subsequent qPCR which was performed as described previously^47^. Transcripts of three housekeeping genes and 32 genes involved in cytokine-, interferon-, chemokine- and innate immunity-related signaling were quantified as fold changes using the ΔΔC_T_-method. For a detailed list of genes and primer sequences, see supplemental Table S2. Transcripts reaching a threshold fold change of 1.5 were regarded as potentially regulated and subjected to RT-qPCR in non-pooled samples.

### Plaque assay

The cerebrum was weighed, diluted in Dulbecco’s modified Eagle Medium (PAA Laboratories) with 50 mg/kg gentamicin (Sigma-Aldrich) to a concentration of 10% and homogenized using Omni Tissue Homogenizer (Süd-Laborbedarf GmbH). Homogenates were serially diluted and added to 6-well culture plates (Sigma-Aldrich) of confluent L cells for 1 hour at room temperature, with gentle horizontal shaking and clockwise rotation in 10 minute intervals. Cells were then covered with methyl cellulose (Sigma-Aldrich) and incubated for 72 hours at 37 °C. Subsequently, methylcellulose was removed and the monolayer was fixed with 10% buffered formalin. Plaques were visualized by staining with crystal violet (Merck). The plaques were counted in wells containing between 10 and 100 plaques and the PFU/ml were determined by multiplying by the dilution factor of the homogenate and dividing by the amount of homogenate added per plate.

### Flow cytometry

For phenotypical analysis of peripheral leukocytes, spleen samples were dissolved to single cell suspension, erythrocytes were lysed and cell numbers were determined as described previously^25^. After blocking the FC-receptor II/III by preincubation with a CD16/CD32-specific Ab (clone 2.4G2; BioXCell), dead cells were stained using the LIVE/DEAD® fixable dead cell stain kit (Invitrogen^TM^ GmbH). Subsequently, CD4- (PacificBlue; clone GK1.5; BioLegend), CD8α- (HV500; clone 53-6.7; BD Biosciences), CD19- (PE-eFluor610; clone 1D3; eBioscience/Thermo Fisher Scientific), CD69- (FITC; clone H1.2F3; BioLegend) and CD44-specific Ab (APC; clone IM7; BioLegend) were added for fluorochrome-conjugated surface marker staining. For intracellular staining of Foxp3 the Foxp3 Transcription Factor Staining Buffer Set (eBioscience/Thermo Fisher Scientific) was used according to the manufacturer’s instructions. Samples were acquired with an LSRII SORP cytometer (BD Biosciences) and analyzed using FlowJo software version 9.6.4 (Tree Star, Ashland, USA). Blood samples were stained according to the same procedure.

### Statistical analysis

All statistical analyzes were conducted using Statistical analysis software SAS 9.3 and the Enterprise Guide 5.1 for Windows (SAS Institute Inc.). Comparison between different groups was performed using multiple Mann-Whitney U tests. Results were considered statistically significant at p-value < 0.05. Box and whisker plots were generated with GraphPad Prism 6.0 (GraphPad Software) and display median, minimum and maximum values as well as upper and lower quartiles. In the RNA Seq experiment, the IL-10 pathway data were also compared between experimental groups by global gene set tests^48^.

### Data Availability

RNA-Seq data can be accessed at GEO/SRA (https://www.ncbi.nlm.nih.gov/geo/query/acc.cgi?acc = GSE103698). All other datasets generated and analysed during the current study are available from the corresponding author on reasonable request.

## Results

### Transcripts of interleukin-10 and related genes are upregulated in the brain during acute Theiler’s murine encephalomyelitis

**Figure 1:**
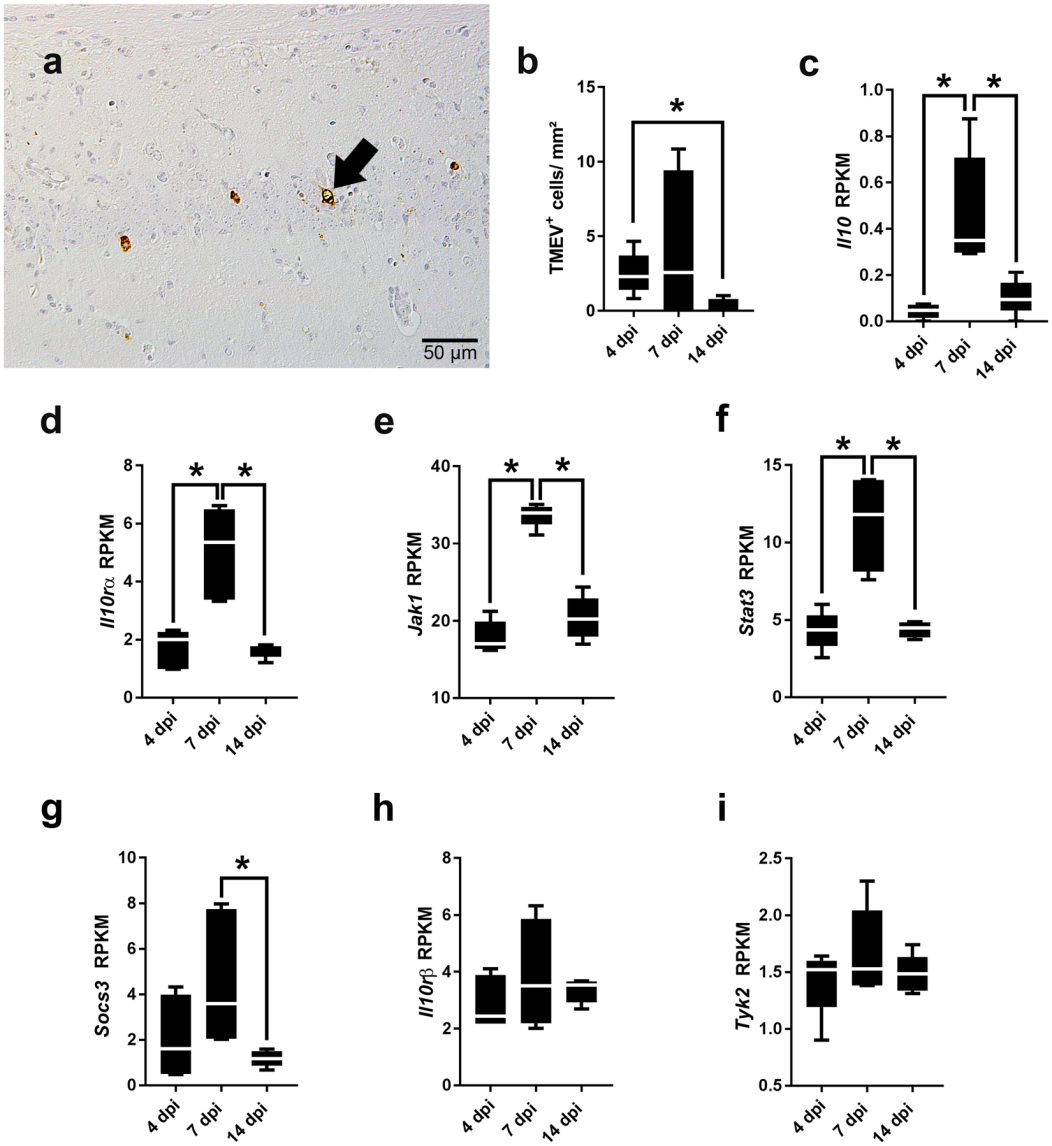
Quantification of Theiler’s murine encephalomyelitis virus (TMEV) antigen and IL-10 pathway genes in the brain of TMEV-infected SJL mice. (**a**) TMEV antigen (arrow) within neurons of the pyramidal layer at 7 days post infection (dpi). TMEV-specific immunohistochemistry. (**b**) Quantification of TMEV^+^ cells in the hippocampus. (**c**–**i**) Gene-wise comparison of *Il10*, *Ill10rα*, *Jak1*, *Stat3*, *Socs3*, *Il10rβ* and *Tyk2* at 4, 7, and 14 dpi. (**b**–**i**) Box plots display median and quartiles with minimum and maximum values. *Significant difference p ≤ 0.05 (Mann-Whitney U test). RPKM = reads per kilobase max. transcript length per million mapped reads.

Intracerebral TMEV infection led to an acute transient polioencephalitis in SJL mice. TMEV antigen was preferentially detectable within neurons of the pyramidal layer but also within the stratum moleculare at 4 and 7 dpi (Fig. 1a). A significant reduction of virus antigen was noted at 14 dpi (p = 0.016; Fig. 1b), indicating virus elimination in the hippocampus of TMEV-infected SJL mice after early infection.

To determine the kinetics of *Il10* and related genes involved in IL-10 downstream signaling during the course of acute TMEV infection, RNA-Seq of brain tissue was performed. Gene-wise comparison showed a significant upregulation of *Il10*, *Il10rα*, *Jak1* and *Stat3* expression at 7 dpi compared to 4 dpi (p = 0.009 for all genes) and a significant downregulation of the same transcripts at 14 dpi compared to 7 dpi (p = 0.009 for all genes; Fig. 1c–f). *Socs3* expression showed similar expression kinetics and a significant difference was detected between 7 and 14 dpi (p = 0.009; Fig. 1g). No significant differences were detected in the expression of *Il10rβ* and *Tyk2* during the infection course (Fig. 1h,i). The pathway-specific global test analysis yielded that the overall expression of IL-10 pathway targets was significantly different between days 4 and 7 (p = 0.002) and between days 7 and 14 (p = 0.004).

### Interleukin-10 receptor blockade enhances hippocampal damage in Theiler’s murine encephalomyelitis virus-infected mice

Transcriptome analysis revealed a transient activation of *Il10* and related transcripts. To elucidate the effect of IL-10 signaling on the course of acute encephalitis, an Ab specifically blocking the IL-10Rα was applied following infection of SJL mice.

**Figure 2:**
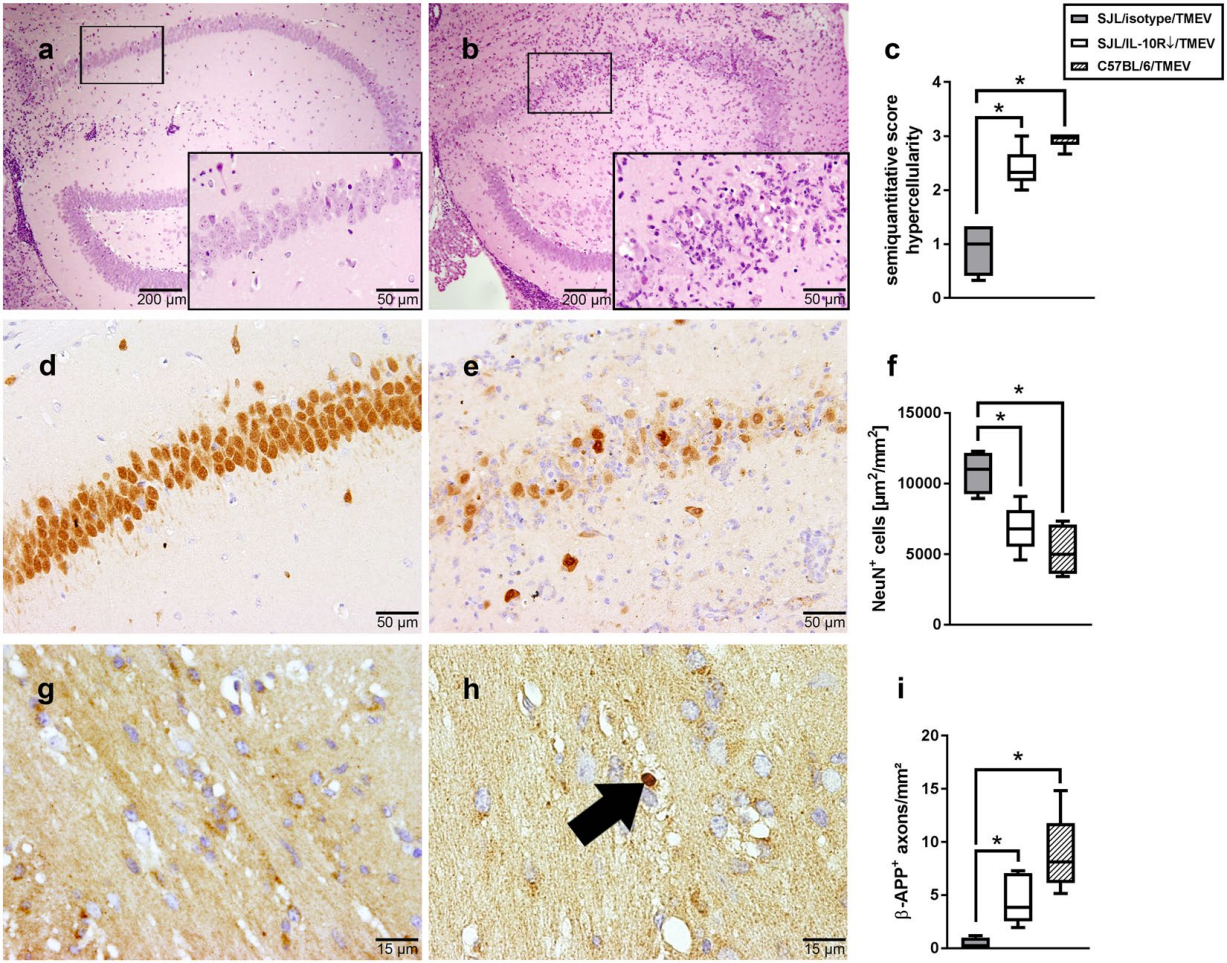
Hippocampal damage in Theiler’s murine encephalomyelitis virus (TMEV)-infected SJL mice following IL-10R blockade resembles lesions in C57BL/6 mice at 7 days post infection. (**a**) Representative image of a hippocampus derived from a TMEV-infected, isotype-treated SJL mouse (control). (**b**) Severe inflammation accompanied by neuronal pyknosis in an infected SJL animal following IL-10R blockade. (**a**,**b**) H&E staining. (**c**) Semiquantitative scoring of hippocampal inflammation. (**d**) NeuN^+^ neurons in an intact pyramidal layer of a TMEV-infected isotype-treated SJL mouse (control). (**e**) Loss of NeuN^+^ neurons following IL-10R blockade in a TMEV-infected SJL animal. (**d**,**e**) NeuN-specific immunohistochemistry (IHC). (**f**) Morphometric analysis of NeuN-specific staining in the hippocampus. (**g**) TMEV-infected, isotype-treated SJL mouse without detectable axonal injury (control). (**h**) Accumulation of β-amyloid precursor protein (β-APP) in a swollen axon (arrow) after TMEV-infection and IL-10R blockade in a SJL mouse. (**g**,**h**) β-APP-specific IHC. (**i**) Quantification of β-APP^+^ axons in the hippocampus and corpus callosum. (**c**,**f**,**i**) Box plots display median and quartiles with minimum and maximum values. *Significant difference p ≤ 0.05 (Mann-Whitney U test).

Histology revealed increased inflammatory responses within the hippocampus of IL-10R blocked SJL mice compared to isotype-treated animals following TMEV infection (p = 0.016). Increased inflammation was associated with neuronal pyknosis (condensation and size reduction of cell body) and loss, preferentially located in the CA1 region (Fig. 2a-c). In accordance with histological alterations, morphometric quantification of NeuN^+^ cells confirmed a loss of mature neurons in the pyramidal cell layer of the hippocampus following anti-IL-10R Ab application (p = 0.032; Fig. 2d–f). Along with neuronal loss, a mild but significant increase of axonal β-APP accumulation, indicative of impaired axonal transport and axonal injury, was found in the hippocampus and adjacent corpus callosum of infected mice following IL-10R blockade compared to isotype-treatment (p = 0.016; Fig. 2g–i). The extent of hippocampal inflammation (histology score), neuronal loss (NeuN) and axonal damage (β-APP) in IL-10R-blocked SJL mice was comparable to the lesions observed in TMEV-infected C57BL/6 mice (Fig. 2c,f,i)

### Augmented polioencephalitis following IL-10R blockade is accompanied by compensatory responses mediated by regulatory T cells and arginase-1 expressing macrophages/microglia

To characterize immune responses in the infected mouse brain following anti-IL-10R Ab application in detail, leukocyte recruitment and glial responses were analyzed in SJL mice and compared to C57BL/6 mice. Additionally, pro- and anti-inflammatory cytokine expression was quantified in SJL mice.

**Figure 3:**
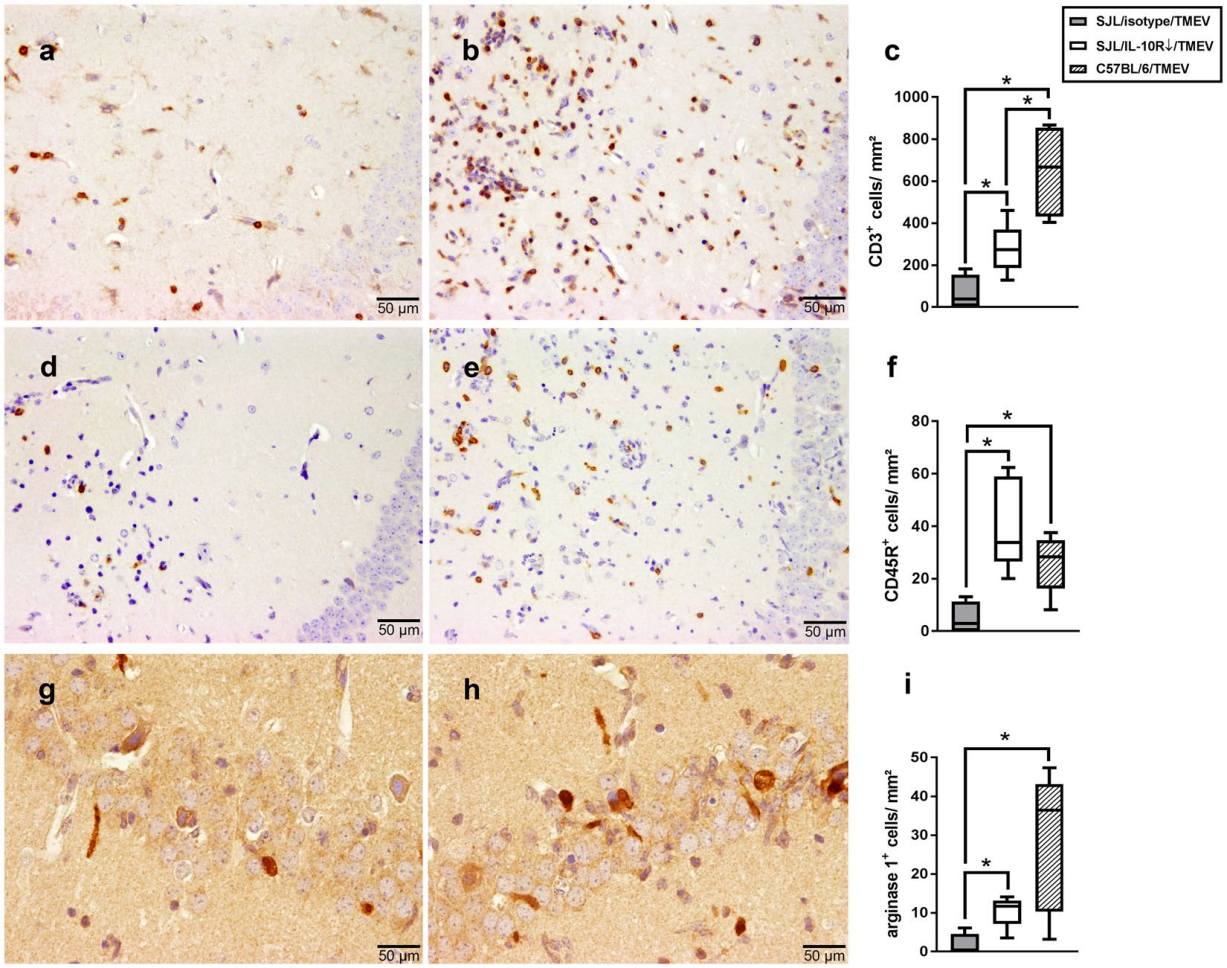
Phenotyping of inflammatory cells in the hippocampus of Theiler’s murine encephalomyelitis virus (TMEV)-infected C57BL/6 and SJL mice with or without IL-10R blockade at 7 days post infection. (**a**) Representative image of an isotype-treated SJL mouse infected with TMEV (control). (**b**) Prominent increase of T cells following IL-10R blockade. (a, b) CD3-specific immunohistochemistry (IHC). (**c**) Quantification of CD3^+^ T cells in the hippocampus. (**d**) Low numbers of CD45R^+^ B cells within the hippocampus of a TMEV-infected isotype-treated SJL animal (control). (**e**) Marked increase of CD45R^+^ B cells after application of anti-IL-10R Ab in a SJL mouse. (**d**,**e**) CD45R-specific IHC. (**f**) Quantification of CD45R^+^ B cells in the hippocampus. (**g**) Few arginase-1 (Arg1)^+^ cells in a TMEV-infected, isotype-treated SJL animal (control). (**h**) Increased numbers of Arg1^+^ cells in the pyramidal cell layer of a SJL mouse after IL-10R blockade. (**g**,**h**) Arg1-specific IHC. (**i**) Quantification of Arg1^+^ macrophages/microglia in the hippocampus. (**c**,**f**,**i**) Box plots display median and quartiles with minimum and maximum values. *Significant difference p ≤ 0.05 (Mann-Whitney U test).

IHC revealed an increased infiltration of CD3^+^ T cells (p = 0.032; Fig. 3a–c) and CD45R^+^ B cells (p = 0.016; Fig. 3d–f) in the hippocampus of TMEV-infected mice following IL-10R blockade. Quantification of CD107b^+^ macrophages/microglia revealed no differences between both groups (supplemental Table S3). However, increased numbers of Arg1^+^ macrophages/microglia were detected in the hippocampus of TMEV-infected, IL-10R blocked mice compared to infected, isotype-treated animals (p = 0.032; Fig. 3g–i). The extent of inflammatory cell infiltration in Ab-treated SJL mice mimics the situation observed in infected C57BL/6 mice. Similar numbers of CD45R^+^ B cells (Fig. 3f) and Arg1^+^ macrophages/microglia (Fig. 3i) were detected in both groups, while increased numbers of CD3^+^ T cells (Fig. 3c) and CD107b^+^ macrophages/microglia (supplemental Table S3) were present in infected C57BL/6 mice.

**Figure 4:**
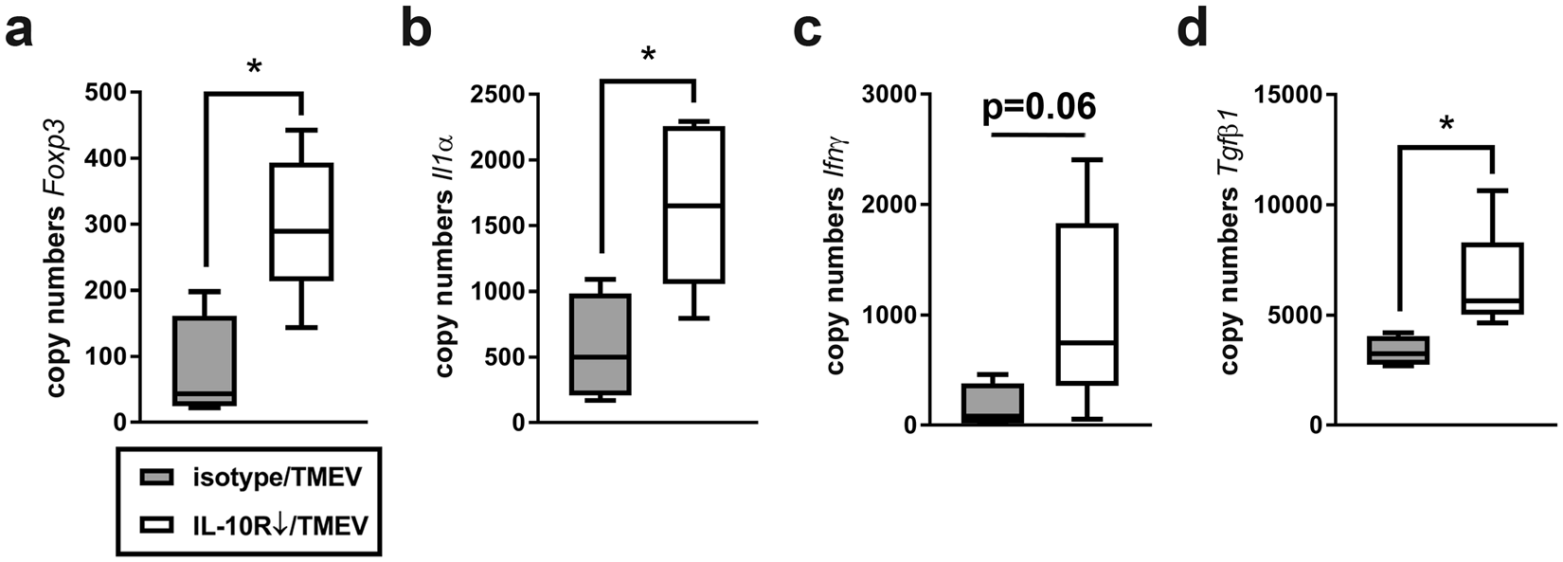
Upregulation of *Foxp3* and cytokine mRNA expression in the brain of Theiler’s murine encephalomyelitis virus (TMEV)-infected SJL mice following IL-10R blockade at 7 days post infection. Quantification of (**a**) *Foxp3*, (**b**) *Il1α*, (**c**) *Ifnγ* and (**d**) *Tgfβ1* mRNA in TMEV-infected animals showing a significant upregulation of three genes in animals treated with anti-IL-10R antibody compared to isotype-treated mice (controls). (**a**–**d**) Box plots display median and quartiles with minimum and maximum values. *Significant difference p ≤ 0.05 (Mann-Whitney U test).

A significantly increased infiltration of Foxp3^+^ Treg (p = 0.032) in the hippocampus together with an elevated Foxp3 mRNA expression, detected by RT-qPCR, was noticed in IL-10R blocked SJL mice compared to isotype-treated controls (p = 0.032; Fig. 4a). Numbers of Foxp3^+^ Treg were also significantly higher in IL-10R-blocked SJL mice compared to C57BL/6 mice (p = 0.016; supplemental Table S3).

For further elucidation of the immunological milieu in the CNS of SJL mice, cytokine expression analyses were performed. RT-qPCR revealed an enhanced expression of *Il1α* mRNA in the infected mouse brain following IL-10R blockade compared to isotype treatment (p = 0.032; Fig. 4b). In addition, the level of *Ifnγ* transcripts was slightly increased following Ab treatment, although the level of significance was not reached (p = 0.064, Fig. 4c). IL-10R blockade also elicited increased mRNA levels of the anti-inflammatory cytokine *Tgfβ1* (p = 0.016; Fig. 4d), indicative of potential compensatory reactions. In contrast, no group differences were determined regarding the expression of *Il2*, *-4*, *-5*, *-6*, *-10* and *Tnf* (supplemental Table S3).

### IL-10R blockade does not reduce virus load in the brain

Since IL-10 is involved in regulation of antiviral immune responses, TMEV antigen distribution and viral RNA copy numbers were quantified in animals receiving IL-10R Ab or isotype control by IHC and RT-qPCR, respectively. IHC showed a preferential infection of hippocampal pyramidal neurons. Contrary to our initial hypothesis of an enhancing effect of IL-10R blockade on antiviral immunity, Ab treatment did not decrease the virus load in the brain. A slight, but non-significant increase of TMEV RNA concentration (p = 0.064) was found in IL-10R-blocked mice compared to non-treated mice (supplemental Figure S1, supplemental Table S3). However, quantification of infectious virus by plaque assay revealed no differences between the groups (supplemental Figure S1, supplemental Table S3).

### IL-10R blockade enhances numbers of splenic chitinase-3-like protein 3 expressing macrophages during acute Theiler’s murine encephalomyelitis

**Figure 5:**
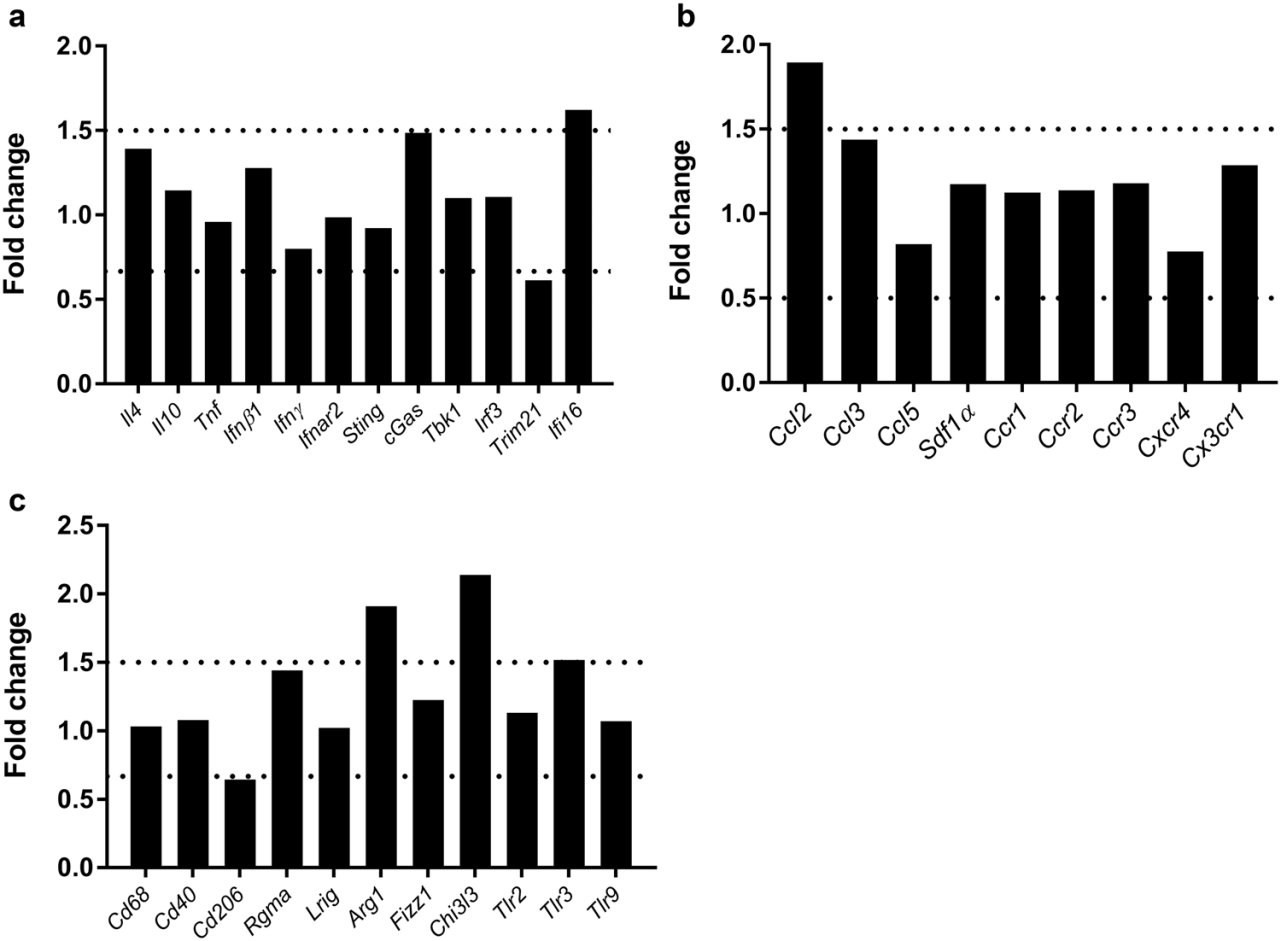
Targeted RT-qPCR based screening approach of pooled spleen samples of Theiler’s murine encephalomyelitis virus (TMEV)-infected SJL mice following IL-10R blockade at 7 days post infection. Fold changes of transcript levels of IL-10R-blocked animals in comparison to the isotype-treated group. Quantification of (**a**) cytokines/interferon-related genes, (**b**) chemokines/chemokine receptors and (**c**) genes related to innate immunity. *Ifi16*, *Ccl2*, *Arg1* and *Chi3l3* reached the 1.5 fold change threshold. For a detailed description of primer sequences and full gene names see Supplemental Table S2.

Effects of IL-10R blockade upon peripheral immune responses were analyzed in SJL mice by flow cytometry and a targeted RT-qPCR based screening approach of pooled spleen samples. RT-qPCR screening in pooled spleen samples was performed for a total number of 32 targets known to be involved in cytokine-, chemokine- and IFN-pathways and innate immune responses. Four of the transcripts were elevated above the threshold of 1.5 fold change in Ab-treated mice compared to isotype-treated controls: *Ifi16*, *Ccl2*, *Arg1*, and *Chi3l3* (Fig. 5).

**Figure 6:**
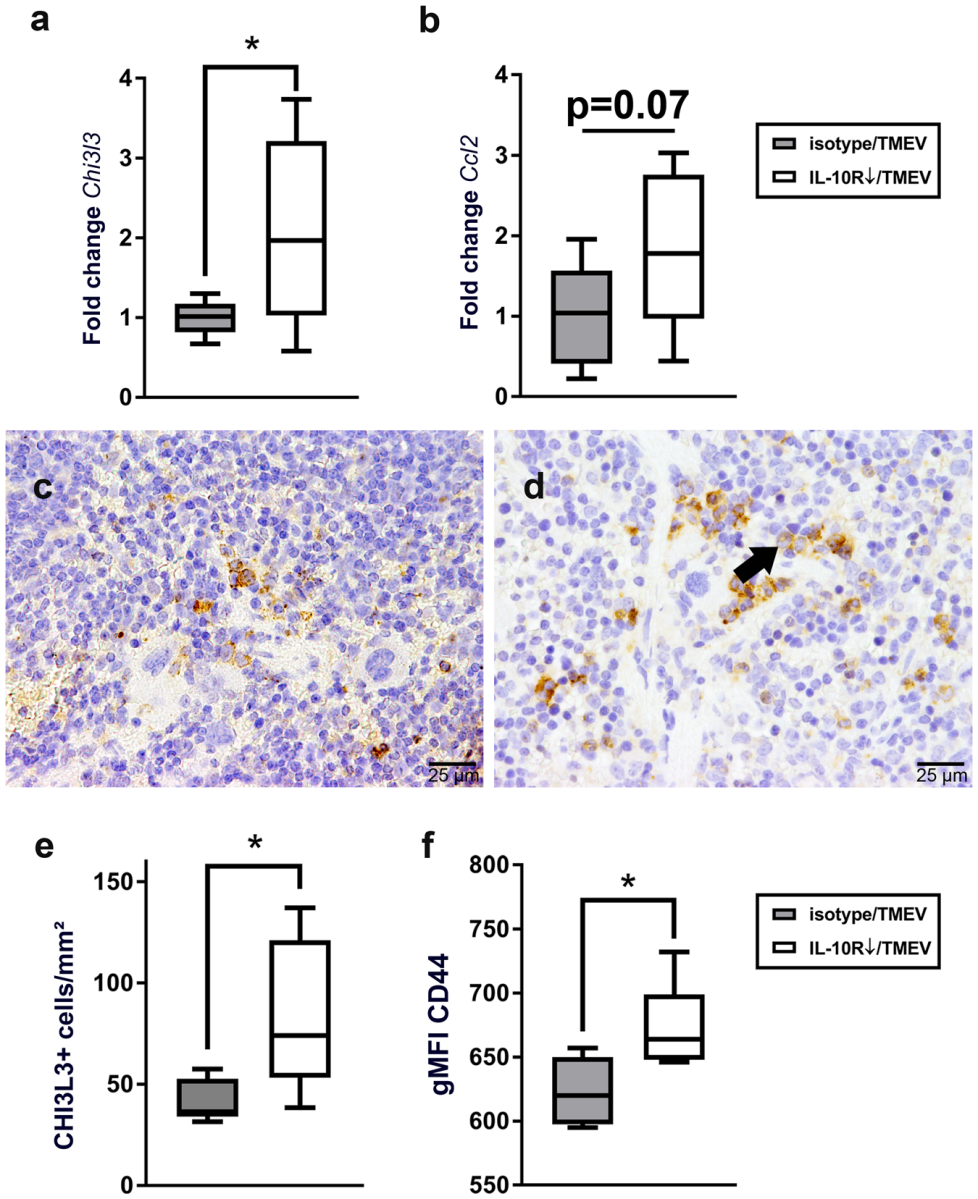
Characterization of the systemic immune response in Theiler’s murine encephalomyelitis virus (TMEV)-infection of SJL mice following IL-10R blockade at 7 days post infection. (**a**) Significant upregulation of *Chi3l3* in TMEV-infected animals receiving anti-IL-10R Ab compared to isotype-treated mice (controls). (**b**) Slight, but non-significant (p = 0.07) upregulation of *Ccl2* in TMEV-infected, IL-10R-blocked animals compared to isotype-treated mice (controls). (**a**,**b**) RT-qPCR analysis of non-pooled (individual) spleen samples. (**c**) Representative image of a spleen from a TMEV-infected, isotype-treated mouse showing low numbers of CHI3L3^+^ cells (control). (**d**) Increased numbers of CHI3L3^+^ cells (arrow) in an animal after application of anti-IL-10R Ab. (**c**,**d**) CHI3L3-specific immunohistochemistry. (**e**) Quantification of CHI3L3^+^ cells in the spleen. (**f**) Significant increase of the geometric mean of fluorescence intensity for CD44 gated on CD8^+^ cytotoxic T cells in the spleen after infection with TMEV and application of IL-10R Ab, determined by flow cytometry. (**a**,**b**,**e**,**f**) Box plots display median and quartiles with minimum and maximum values. *Significant difference p ≤ 0.05 (Mann-Whitney U test).

Accordingly, non-pooled samples of individual animals were used for quantification of *Ifi16*, *Ccl2*, and *Chi3l3* by RT-qPCR. *Arg1* was not included due to overlapping biological functions of *Arg1* and *Chi3l3*. Testing of single samples revealed a significant increase of *Chi3l3* mRNA in IL-10R-blocked animals (p = 0.022; Fig. 6a). In addition, a mildly increased expression of *Ccl2* mRNA, which did not reach the level of significance (p = 0.070), was found in Ab treated mice (Fig. 6b). No difference between groups was found for the expression of *Ifi16* (Supplemental Table S3). To confirm the increased expression of *Chi3l3* mRNA on the protein level, spleen tissue was further examined by IHC. In line with the results obtained by RT-qPCR, an increased accumulation of CHI3L3^+^ cells with a macrophage-like morphology was observed in spleen tissue of IL10R-blocked mice (p = 0.032; Fig. 6c–e).

Systemic immune responses were further analyzed by flow cytometry of spleen and blood samples using markers for B cells (CD19), cytotoxic T cells (CD8), T helper cells (CD4), Treg (Foxp3) and activated T cells (CD44, CD69). In accordance with the observed mild expressional changes, flow cytometry revealed only minimal variations between IL-10R-blocked mice and isotype treated controls. A significant upregulation of CD44 expression on CD8^+^ T cells was observed in spleens of IL-10R-blocked animals (p = 0.037; Fig. 6f), but not in peripheral mononuclear blood cells (Supplemental Table S3). In contrast, no differences were detectable regarding the overall expression of CD19, CD4, CD8 and Foxp3 on peripheral mononuclear blood cells and cells derived from spleen tissue. The gMFI of CD69^+^CD8^+^ and CD69^+^CD4^+^ cells as well as the gMFI of CD44^+^CD4^+^ cells also did not display any differences between both groups (Supplemental Table S3, Supplemental Figure S2).

In summary, IL-10R neutralization led to limited transcriptional and phenotypical changes in the peripheral immune system during acute TMEV infection. However, increased numbers of splenic CHI3L3^+^ macrophages were detected in treated mice, indicative of immunomodulatory responses.

## Discussion

IL-10 exhibits profound modulatory effects and critically controls the balance of host immune responses^9,10^. The present study highlights the importance of IL-10R signaling for immune regulation and prevention of CNS damage following acute neurotropic virus infection.

TMEV-infection of SJL mice resulted in a transient polioencephalitis with TMEV-antigen detection in the hippocampus peaking at 7 dpi. In parallel, RNA-Seq analyzes revealed a transiently enhanced expression of *Il-10* and related genes, such as *Il10rα*, *Jak1*, *Stat3*, *and Socs3*. IL-10 signals through the IL-10R, a transmembrane protein composed of an α- and β-subunit. Whereas IL-10Rβ is constitutively present on most cell types, IL-10Rα is mainly expressed on immune cells, such as lymphocytes, dendritic cells, macrophages, and microglia, thus being rapidly upregulated in inflammatory conditions as found in the present study^9,49,50^. Upon binding, activation of the receptor-associated tyrosine kinase JAK1 leads to STAT3 activation. STAT3 binds to promotors of IL-10 responsive genes and enhances *Socs3* transcription, which triggers anti-inflammatory immune responses^9,51,52^.

In order to elucidate the functional significance of this cytokine pathway in neurotropic virus infection, the α-subunit of IL-10R was selectively blocked in TMEV-infected SJL mice. IL-10R neutralization considerably triggered inflammation of the hippocampus with an enhanced loss of NeuN^+^ mature neurons and increased β-APP expression of injured axons, indicative of neuroprotective properties of intact IL-10 signaling in SJL mice. Interestingly, the observed hippocampal pathology and inflammation closely resemble the lesions detected in infected C57BL/6 mice in quality and quantity which supports the notion that differential IL-10 signaling contributes to the divergent disease phenotypes observed between SJL and C57BL/6 mice in TME^26^. TMEV-infection of C57BL/6 mice has become a valuable model to investigate hippocampal damage and infection-induced epilepsy^2,3,5,53,54^. However, neither manifest behavioral changes nor seizures were observed in the present study, which might be attributed to a comparatively lower extent of neuropathology and neuronal dysfunction. The BeAn strain used here has been shown to cause seizures less frequently compared to more neurovirulent strains, such as the DA-strain^5,55^. Targeted methods such as video/EEG monitoring and specific behavioral tests are needed to identify subtle clinical changes and the clinical relevance of enhanced hippocampal damage in SJL mice following IL-10R blockade.

Similar to other picornaviruses, TMEV is capable of inducing apoptosis of infected cells^56,57^. In addition, apoptosis of non-infected cells was observed in the hippocampus of TMEV-infected C57BL/6 mice as well, indicating virus-independent mechanisms of neuronal death triggered by pro-inflammatory responses (bystander injury)^58^. In line with these observations, neuronal damage was not associated with increased viral load in this study. IL-10 signaling has been shown to reduce neuronal apoptosis and support blood brain barrier integrity following traumatic CNS injury in rodent models^59,60^. IL-10 also lessens neurotoxicity induced by lipopolysaccharide or oxygen-glucose deprivation and sustains neuronal function by inducing neurotrophic factors *in vitro*^61,62^. Moreover, sound IL-10 signaling protects from CNS damage mediated by encephalitogenic Th17 cells as demonstrated in mice infected with a mosquito-borne alphavirus^63^.

IL-6 and TNF as well as infiltrating macrophages and activated microglia are crucially involved in neuronal damage and seizure induction in C57BL/6 mice^64–67^. Unlike the situation seen in this mouse strain^5,68^, increased neuronal damage in our study was not associated with an up-regulation of *Il6* and *Tnf*, suggesting that neuronal death might be attributed to other mechanisms in SJL mice. Increased hippocampal damage following disturbed IL10 signaling was associated with sequestration of CD3^+^ T cells and CD45R^+^ B cells and increased expression of *Il1α mRNA*. IL-1α represents a pro-inflammatory acute phase cytokine which is produced by macrophages and microglia and released following viral infection and CNS injury^69,70^. IL-1α induces neurotoxic reactive astrocytes (A1 astrocytes), which contribute to neuron and oligodendrocyte death in neurodegenerative diseases, such as Alzheimer’s disease, amyotrophic lateral sclerosis and multiple sclerosis^71^. IL-1R activation has also been shown to induce excitotoxicity and neuronal death by excessive glutamate production in a murine model of HIV-1 encephalitis^72^. Other potential mechanisms to trigger neurodegeneration include cytokine-mediated increase of the blood brain barrier permeability with accelerated leukocyte influx and cerebral edema^73,74^.

In contrast to an improved efficacy of antiviral responses following IL-10R blockade observed in certain other infectious CNS disease models^17,18,75,76^, a reduction of cerebral virus load was not detectable in our experimental setting. Previous studies suggested that IL-10 overexpression in TMEV-infected SJL mice might represent a prerequisite for viral persistence^26,27^. However, the present data clearly indicate that IL-10-mediated responses - at least during the acute infection phase - do not account for insufficient antiviral immunity of SJL mice in the TME model.

Simultaneously to pro-inflammatory CNS responses, an accelerated recruitment of Foxp3^+^ Treg together with an elevated *Foxp3* and *Tgfβ1* expression were observed in the brain. This might represent compensatory reactions aiming to prevent collateral tissue damage and maintain cerebral immune homeostasis, as observed in a variety of autoimmune, infectious, traumatic, and neurodegenerative disorders^77–80^. Notably, neuronal *Tgfβ1* expression in the hippocampus also correlates with neuronal pyknosis in TMEV-infected C57BL/6 mice, representing a mechanism to prevent neurodegeneration^3^. However, besides its neuroprotective properties, the anti-inflammatory cytokine *Tgfβ* has been shown to impair antiviral immunity. For instance, in mice infected with the high virulent GDVII TMEV strain, prominent *Tgfβ* expression is supposed to reduce T cell responses which in turn prevent tissue damage but might result in fatal outcome by reducing antiviral immunity^81^. *Tgfβ* also supports Foxp3^+^ Treg recruitment and immunomodulatory properties of macrophages/microglia in an autocrine and paracrine manner^82–84^. Moreover, enhanced Treg-differentiation has been described following neuronal damage to reduce CNS injury and neuroinflammation^85^. Similarly, immunomodulatory macrophages/microglia and Foxp3^+^ Treg protect from EAE through deactivation of encephalitogenic Th1 and Th17 cells^86,87^. However, immunomodulatory macrophages/microglia have also the ability to dampen antiviral immunity by restricting pro-inflammatory and CD4^+^ T cell responses, as described for human cytomegalovirus infection^88^. Similarly, Treg reduce antiviral immunity in TMEV-infected SJL mice. Functional inactivation of Treg by application of anti-CD25-Ab prior to TMEV-infection results in enhanced virus-specific immunity, reduced viral load, and delayed disease progression^89^. Equally, adoptive transfer of Treg in the acute disease phase leads to disease exacerbation in TMEV-infected SJL mice^90^.

In peripheral lymphoid organs, molecular analyzes and flow cytometry revealed rather limited effects of IL-10R blockade on adaptive immune responses under acute infectious conditions. Although cytotoxic T cells are crucial for TMEV elimination in C57BL/6 mice^54,91,92^, CD8 activation following IL-10R blockade as shown by heightened CD44 expression on splenic CD8^+^ T cells was obviously unable to control brain infection in SJL mice. In accordance with this, no effects of IL-10R neutralization on the virus load within the CNS were observed in our previous study investigating advanced TME, even though profound cytokine and CD4- and CD8-mediated responses were detected in lymphoid organs^25^. *Chi3l3* gene expression together with CHI3L3^+^ cell accumulation in the spleen indicates enhanced immunomodulatory properties of splenic macrophages in Ab treated mice^32^. Similar to the proposed situation in the brain, expression of CHI3L3in the spleen may represent a counter-regulatory attempt to maintain peripheral immune homeostasis and limit systemic immunopathology in IL-10R-blocked mice.

In conclusion, the present study reveals neuroprotective properties of intact IL-10R signaling and highlights the importance of the IL-10 pathway in maintaining hippocampal integrity in SJL mice following TMEV infection. IL-10R blockade causes severe neuronal damage in SJL mice, mimicking brain lesions observed in seizure-prone C57BL/6 mice. Since intervention in IL-10 signaling is considered as a novel therapeutic approach in viral and immune mediated disorders^93,94^, the presented data illustrate the potential risk of disease exacerbation after IL-10R neutralization in CNS disorders which are caused by or developed in parallel with neurotropic virus infection. Future studies are needed to further elucidate the mechanisms involved in IL-10-mediated neuroprotection in the TME model.

## Electronic supplementary material

Supplementary Information
