# Supplementary material for: Intact interleukin-10 receptor signaling protects from hippocampal damage elicited by experimental neurotropic virus infection of SJL mice
Source: Sci Rep. 2018 Apr 17;8:6106. doi: 10.1038/s41598-018-24378-z (PMC5904160; doi:10.1038/s41598-018-24378-z)
Supplement: Supplementary file 1 — Supplementary Information [file 41598_2018_24378_MOESM1_ESM.pdf]

Intact interleukin-10 receptor signaling protects from hippocampal damage elicited by experimental neurotropic virus infection of SJL mice

Ann-Kathrin Uhde[1‡], Malgorzata Ciurkiewicz[1,2‡], Vanessa Herder[1,2], Muhammad Akram Khan[1,2,3], Niko Hensel[4], Peter Claus[2,4,5], Michael Beckstette[6], René Teich[6], Stefan Floess[6], Wolfgang Baumgärtner[1,2,5], Klaus Jung[7], Jochen Huehn[6#] and Andreas Beineke[1,2*#]

[1] Department of Pathology, University of Veterinary Medicine Hannover, Hannover, Germany

[2] Center for Systems Neuroscience, Hannover, Germany

[3] Department of Pathobiology, Faculty of Veterinary & Animal Sciences, PMAS–Arid Agriculture University, Rawalpindi, Pakistan

[4] Institute of Neuroanatomy and Cell Biology, Hannover Medical School, Hannover, Germany

[5] Niedersachsen-Research Network on Neuroinfectiology (N-RENNT), Hannover, Germany.

[6] Experimental Immunology, Helmholtz Centre for Infection Research, Braunschweig, Germany

[7] Institute for Animal Breeding and Genetics, University of Veterinary Medicine Hannover, Hannover, Germany

[#] J. Huehn and A. Beineke contributed equally to this work

[‡] A.-K. Uhde and M. Ciurkiewicz contributed equally to this work

[*] Corresponding author:

Prof. Dr. Andreas Beineke
Department of Pathology, University of Veterinary Medicine Hannover
Buenteweg 17
30559 Hannover
Germany
Phone number: 00495119538640
Fax number: 00495119538675
Email address: Andreas.Beineke@tiho-hannover.de

Supplemental table S1

Summary of primer pairs used for RT-qPCR (brain)

	Gene	Primer sequence		length
Interleukin-1α	*Il1α*	F	AAG CAA CGG GAA GAT TCT GA	179 bp
		R	TGA CAA ACT TCT GCC TGA CG	
Interleukin-2	*Il2*	F	GCA GGA TGG AGA ATT ACA GGA	183 bp
		R	TGA AAT TCT CAG CAT CTT CCA A	
Interleukin-4	*Il4*	F	CCT CAC AGC AAC GAA GAA CAC C	133 bp
		R	CAT CGA AAA GCC CGA AAG AGT C	
Interleukin-5	*Il5*	F	ATG GAG ATT CCC ATG AGC AC	180 bp
		R	CCC ACG GAC AGT TTG ATT CT	
Interleukin-6	*Il6*	F	GTT CTC TGG GAA ATC GTG GA	176 bp
		R	CCA GAG GAA ATT TTC AAT AGG C	
Interleukin-10	*Il10*	F	CCA AGC CTT ATC GGA AAT GA	162 bp
		R	TTT TCA CAG GGG AGA AAT CG	
Forkhead box P3	*Foxp3*	F	TTC-TCA-CAA-CCA-GGC-CAC-TTG	88 bp
		R	CCC-AGG-AAA-GAC-AGC-AAC-CTT	
Tumor necrosis factor	*Tnf*	F	GCC TCT TCT CAT TCC TGC TT	203 bp
		R	CAC TTG GTG GTT TGC TAC GA	
Interferon-γ	*Ifnγ*	F	CAC GGC ACA GTC ATT GAA AG	144 bp
		R	AAT CTG GCT CTG CAG GAT TT	
Transforming growth factor-β1	*Tgfβ1*	F	TTG CTT CAG CTC CAC AGA GA	183 bp
		R	TGG TTG TAG AGG GCA AGG AC	
Theiler's murine encephalomyelitis virus	*TMEV*	F	GAC TAA TCA GAG GAA CGT CAG C	129 bp
		R	GTG AAG AGC GGC AAG TGA GA	
glyceraldehyde-3-phosphate dehydrogenase	*Gapdh*	F	GAG GCC GGT GCT GAG TAT GT	288 bp
		R	GGT GGC AGT GAT GGC ATG GA	
hypoxanthine guanine phosphoribosyl transferase	*Hprt*	F	GGA CCT CTC GAA GTG TTG GA	169 bp
		R	TTG CGC TCA TCT TAG GCT TT	
β-Aktin	*Actβ*	F	GGC TAC AGC TTC ACC ACC AC	233 bp
		R	ATG CCA CAG GAT TCC ATA CC	

bp = base pairs; F = forward; R = reverse

Supplemental table S2

Summary of primer pairs used for targeted RT-qPCR screening (spleen)

Gene name	Gene symbol	mRNA NCBI reference sequence	Primer sequence	
Interleukin 4	*Il4*	NM_021283.2	F	ATTCATCGATAAGCTGCAC
			R	GTACTACGAGTAATCCATTTGC
Interleukin 10	*Il10*	NM_010548.2	F	TGATTTTAATAAGCTCCAAGAC
			R	TGTTTTAGCTTTTCATTTTGAT
Tumor necrosis factor	*Tnf*	NM_013693.3	F	TCAGATCATCTTCTCAAAATTC
			R	ACTAGTTGGTTGTCTTTGAGAT
Interferon β1	*Ifnβ1*	NM_010510.1	F	GACAGTACTAGAGGAAAAGCAA
			R	AGCTGTTGTACTTCATGAGTTT
Interferon γ	*Ifnγ*	NM_008337.3	F	CTATTTTAACTCAAGTGGCATAG
			R	TTCAAAGAGTCTGAGGTAGAAA
Interferon alpha and beta receptor subunit 2	*Ifnar2*	XM_006522923.2	F	ATGAACCTTGCACTATAAACAT
			R	GTCTTTGCTCATGACTGTGTA
Stimulator of interferon genes	*Sting*	NM_028261.1	F	GTCTAGGAAGCAGAAGATGC
			R	CAGAAAGATGAGGGCTACATA
Cyclic GMP-AMP synthase	*cGas*	NM_173386.5	F	GTATTATCAGCTACCAAGATGC
			R	TCCACACTGACATCTATATCTTT
TANK binding kinase	*Tbk1*	NM_019786.4	F	GAACTGGTTAAGGATGATTACA
			R	CAACTTCTCATACACTTTCACA
Interferon regulatory factor 3	*Irf3*	NM_016849.4	F	TACACTCTGTGGTTCTGCAT
			R	ACATGTAGGAACAACCTTGAC
Tripartite motif containing 21	*Trim21*	NM_001082552.2	F	GAGGACCCTGGTTAGATTC
			R	ATACTCATAGGCTCCACCAT
Interferon gamma inducible protein 16	*Ifi16*	NM_008329.2	F	GAGATCAGAAGTAACAGGAGAA
			R	GCTTGTAGTTGATGTAGGTGTT
C-C motif chemokine ligand 2	*Ccl2*	NM_011333.3	F	AAGAAGCTGTAGTTTTTGTCAC
			R	AGGTTTTTAATGTATGTCTGGA
C-C motif chemokine ligand 3	*Ccl3*	NM_011337.2	F	GTGTCATTTTCCTGACTAAGAG
			R	AGTTCCAGGTCAGTGATGTAT
C-C motif chemokine ligand 5	*Ccl5*	NM_013653.3	F	CAATCTTGCAGTCGTGTTT
			R	TATCCTAGCTCATCTCCAAATA
Stromal cell-derived factor 1 alpha	*Sdf1α*	NM_013655.4	F	GTTCTTCCATTTGTGTACTCTG
			R	GACTCCTGGTTTAGCTGATAG

Supplemental table S2: continued

**Gene name**	**Gene symbol**	**mRNA NCBI reference sequence**	**Primer sequence**	
C-C chemokine receptor 1	*Ccr1*	NM_009912.4	F	CTTGAACCTTGAATTCATAAAG
			R	AAAGACAGTGAGTCTGTGTTTC
C-C chemokine receptor 2	*Ccr2*	NM_009915.2	F	ATAAAGGAGCCATACCTGTAAA
			R	CATATTATTGTCTTCCATTTCC
C-C chemokine receptor 3	*Ccr3*	NM_009914.4	F	ATGATGTTTACTACCTGACTGG
			R	TCTACTTGTCTCTGGTGAATTT
C-X-C motif chemokine receptor 4	*Cxcr4*	NM_009911.3	F	AGGTAGCAGTGAAACCTCTG
			R	AAGTGTATATACTCACACTGATCG
Chemokine (C-X3-C motif) receptor 1	*Cx3cr1*	NM_009987.4	F	ACGGTGTCACCATTAGTCT
			R	CTTTCTCTTTGTGAACATGAAC
CD68 molecule	*Cd68*	NM_001291058.1	F	GATTCAAACAGGACCTACATC
			R	CTCTCGAAGAGATGAATTCTG
CD40 molecule	*Cd40*	NM_011611.2	F	ATGTCATCTGTGGTTTAAAGTC
			R	CTTGACCACCTTTTTGATATAG
CD206 molecule	*Cd206*	NM_008625.2	F	TGGTTATCCAAATAACTTCATC
			R	CAAAAATTTTAAAGCACTTGTT
Repulsive guidance molecule family member a	*Rgma*	NM_177740.5	F	CTCTCATCGACAATAATTACCT
			R	GAAGATGATGGTGAGCTTG
Leucine-rich repeats and immunoglobulin-like domains 1	*Lrig1*	NM_008377.2	F	ATCACTGATGTGAAAATAGACG
			R	AGGATGGAGTTTCTAAGACTGT
Arginase1	*Arg1*	NM_007482.3	F	AGCCAAAGTCCTTAGAGATTAT
			R	CTCACGTCATACTCTGTTTCTT
Resistin like alpha	*Fizz1*	NM_020509.3	F	GATGAAGACTACAACTTGTTCC
			R	AGGGATAGTTAGCTGGATTG
Chitinase 3-like 3	*Chi3l3*	NM_009892.2	F	GAATCTGTGGAGAAAGACATT
			R	TAGTCAAGAGACTGAGACAGTT
Toll-like receptor 2	*Tlr2*	NM_011905.3	F	CAGGATCTTGTTTCTGAGTGT
			R	CTGAGATTTGACGCTTTGT
Toll-like receptor 3	*Tlr3*	NM_126166.4	F	AGAAGATAAAGCGAGTTTCAC
			R	AAGCCAAGTACATTCTGATTT
Toll-like receptor 9	*Tlr9*	NM_031178.2	F	CTCTCCATACACTGAACTCTTC
			R	CATAGAGAAAGTTCTCGCTTAG

F = forward; R = reverse

Supplemental table S3

Summary of statistical analyzes: IL-10R blockade during Theiler's murine encephalomyelitis virus (TMEV) infection at 7 days post infection; p-values			
**Investigation**	**TMEV-infected SJL mice with and without IL-10R blockade**	**TMEV-infected C57BL/6 and SJL mice**	**TMEV-infected C57BL/6 mice and SJL mice with IL-10R blockade**
**Histology (H&E)**			
Hypercellularity (semiquantitative)	↑**0.016***	↑**0.016***	0.056
**Immunohistochemistry, hippocampus**			
CD3	↑**0.032***	↑**0.016***	↑**0.016***
CD45R	↑**0.016***	↑**0.032***	0.222
Foxp3	↑**0.032***	0.413	↓**0.016***
CD107b	0.1111	↑**0.016***	↑**0.032***
Arginase-1	↑**0.032***	↑**0.032***	0.151
β-APP	↑**0.016***	↑**0.016***	0.056
NeuN	↓**0.032***	↓**0.016***	0.222
TMEV	0.921	0.286	0.310
**Immunohistochemistry, spleen**			
CHI3L3	↑**0.032***	n.d.	n.d.
**RT-qPCR (mRNA), hippocampus**			
Il1α	↑**0.032***	n.d.	n.d.
Il2	0.518	n.d.	n.d.
Il4	1.000	n.d.	n.d.
Il5	0.161	n.d.	n.d.
Il6	0.531	n.d.	n.d.
Il10	0.600	n.d.	n.d.
Ton	0.064	n.d.	n.d.
Tgfβ	↑**0.016***	n.d.	n.d.
Ifnγ	0.064	n.d.	n.d.
Foxp3	↑**0.032***	n.d.	n.d.
TMEV	0.064	n.d.	n.d.
**TMEV Plaque assay, cerebrum**			
PFU/ml	0.905	n.d.	n.d.
**RT-qPCR (mRNA), single samples, spleen**			
Chi3l3	↑**0.022***	n.d.	n.d.
Ccl2	0.070	n.d.	n.d.
Ifi16	0.263	n.d.	n.d.
**Flow cytometry, blood**			
$CD4^+$ [%]	1.0000	n.d.	n.d.
$CD8^+$ [%]	0.753	n.d.	n.d.
$CD19^+$ [%]	1.000	n.d.	n.d.
$CD4^+$ $Foxp3^+$ [%]	0.835	n.d.	n.d.
gMFI CD69 gated on $CD4^+$ cells	0.531	n.d.	n.d.
gMFI CD69 gated on $CD8^+$ cells	0.531	n.d.	n.d.
gMFI CD44 gated on $CD4^+$ cells	0.676	n.d.	n.d.
gMFI CD44 gated on $CD8^+$ cells	1.000	n.d.	n.d.

Supplemental table S3: continued

**Summary of statistical analyzes: IL-10R blockade during Theiler's murine encephalomyelitis virus (TMEV) infection at 7 days post infection; p-values**			
**Investigation**	**TMEV-infected SJL mice with and without IL-10R blockade**	**TMEV-infected C57BL/6 and SJL mice**	**TMEV-infected C57BL/6 mice and SJL mice with IL-10R blockade**
**Flow cytometry, spleen**			
$CD4^+$ [%]	0.210	n.d.	n.d.
$CD8^+$ [%]	0.403	n.d.	n.d.
$CD19^+$ [%]	0.296	n.d.	n.d.
$CD4^+$ $Foxp3^+$ [%]	1.000	n.d.	n.d.
gMFI CD69 gated on $CD4^+$ cells	0.144	n.d.	n.d.
gMFI CD69 gated on $CD8^+$ cells	0.210	n.d.	n.d.
gMFI CD44 gated on $CD4^+$ cells	0.144	n.d.	n.d.
gMFI CD44 gated on $CD8^+$ cells	↑**0.037***	n.d.	n.d.

↑↓ = significant up(↑)- or downregulation (↓) in first versus the second group; * bold p-values = significant difference between both groups, $p \leq 0.05$, Mann-Whitney U tests. β-APP = β-amyloid precursor protein; PFU = Plaque forming units; gMFI = geometric mean of fluorescence intensity; H&E = hematoxylin and eosin staining; NeuN = neuron-specific nuclear protein; n.d.= not determined

Supplemental figures

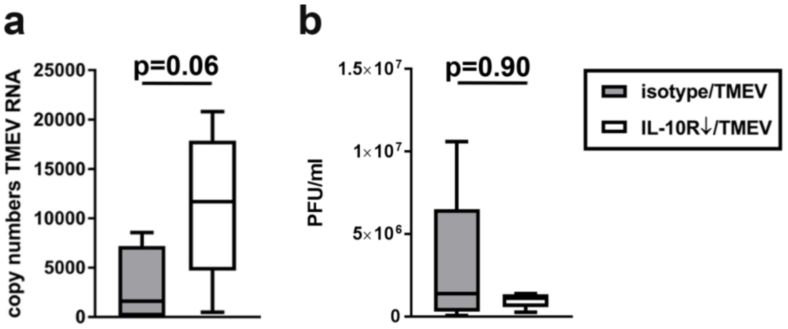

Supplemental figure S1: quantification of Theiler's murine encephalomyelitis virus (TMEV) in the brain. (**a**) Quantification of viral RNA by RT-qPCR. (**b**) Quantification of infectious virus by plaque assay. Statistical analyzes reveal a slight, non-significant increase of TMEV RNA in animals with IL-10R blockade compared to TMEV-infected isotype-treated animals at 7 days post infection. However, no differences are detected in the quantification of infectious virus particles. Box plots display median and quartiles with minimum and maximum values. PFU = Plaque forming units.

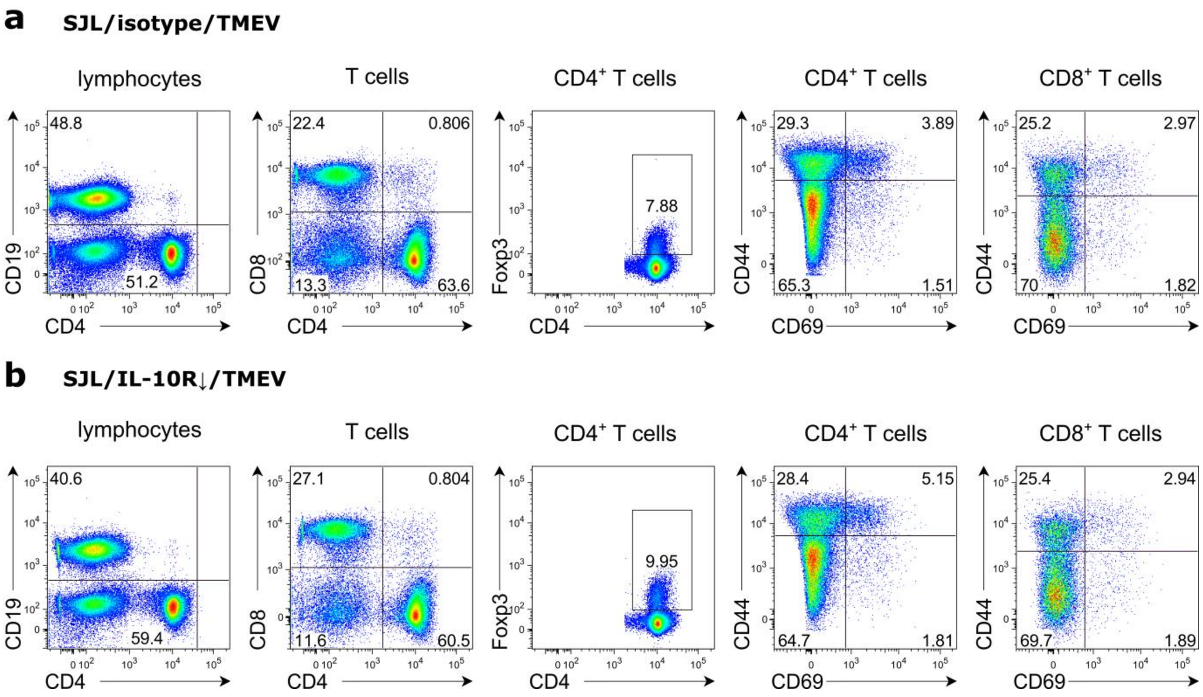

Supplemental figure S2: Flow cytometry analysis of Theiler's murine encephalomyelitis virus (TMEV)-infected SJL mice following IL-10R blockade at 7 days post infection. Representative dot plots of an isotype-treated animal (control) (**a**) and an IL-10R-blocked animal (**b**). No significant differences were detected in the percentages of investigated cell subsets including $CD19^+$ B cells, $CD4^+$ T cells and $CD8^+$ T cells, as well as expression of CD44 and CD69 on $CD4^+$ T cells and $CD8^+$ T cells, respectively.
